# Effects of the Diet on the Microbiota of the Red Palm Weevil (Coleoptera: Dryophthoridae)

**DOI:** 10.1371/journal.pone.0117439

**Published:** 2015-01-30

**Authors:** Matteo Montagna, Bessem Chouaia, Giuseppe Mazza, Erica Maria Prosdocimi, Elena Crotti, Valeria Mereghetti, Violetta Vacchini, Annamaria Giorgi, Alessio De Biase, Santi Longo, Rita Cervo, Giuseppe Carlo Lozzia, Alberto Alma, Claudio Bandi, Daniele Daffonchio

**Affiliations:** 1 Dipartimento di Scienze Agrarie e Ambientali, Università degli Studi di Milano, Milano, Italy; 2 Dipartimento di Scienze per gli Alimenti, la Nutrizione, l’Ambiente, Università degli Studi di Milano, Milano, Italy; 3 Consiglio per la ricerca e la sperimentazione in agricoltura, Research Centre for Agrobiology and Pedology, Cascine del Riccio, Italy; 4 Dipartimento di Scienze Veterinarie e Sanità Pubblica, Università degli Studi di Milano, Milano, Italy; 5 Dipartimento di Biologia e Biotecnologie “C. Darwin”, Università degli Studi di Roma “La Sapienza”, Roma, Italy; 6 Dipartimento di Gestione dei Sistemi Agroalimentari e Ambientali, Università di Catania, Catania, Italy; 7 Dipartimento di Biologia, Università degli Studi di Firenze, Firenze, Italy; 8 Dipartimento di Scienze Agrarie, Forestali e Alimentari, Università di Torino, Grugliasco, Italy; 9 Biological and Environmental Sciences and Engineering Division, King Abdullah University of Science and Technology, Thuwal, Kingdom of Saudi Arabia; International Atomic Energy Agency, AUSTRIA

## Abstract

*Rhynchophorus ferrugineus*, also known as the red palm weevil, is regarded as the major pest of palm trees. Although studies of the microbiota associated with this species have been performed in recent years, little attention has been dedicated to the influence of the diet in shaping the host bacterial community. Here, we investigated the influence of food sources (i.e. palm tissues *vs* apple based substrate) on the microbial diversity associated with RPW, which was compared with the microbiota associated with wild individuals of the sister species *Rhynchophorus vulneratus*. The bacterial characterization was performed using a culture independent approach, i.e. the 16S rRNA pyrotag, and a culture dependent approach for a subset of the samples, in order to obtain bacterial isolates from RPW tissues. The bacterial community appeared significantly influenced by diet. Proteobacteria resulted to be the most abundant clade and was present in all the specimens of the three examined weevil groups. Within Proteobacteria, Enterobacteriaceae were identified in all the organs analysed, including hemolymph and reproductive organs. The apple-fed RPWs and the wild *R. vulneratus* showed a second dominant taxon within Firmicutes that was scarcely present in the microbiota associated with palm-fed RPWs. A comparative analysis on the bacteria associated with the palm tissues highlighted that 12 bacterial genera out of the 13 identified in the plant tissues were also present in weevils, thus indicating that palm tissues may present a source for bacterial acquisition.

## Introduction

The Red Palm Weevil (hereafter RPW), *Rhynchophorus ferrugineus* Olivier (Coleoptera; Dryophthoridae), is regarded today as the major pest of palm, attacking over 20 palm species belonging to 16 different genera worldwide [1]. RPW is native to South Eastern Asia, but due to the international exchange of infected plant material, during the last two decades it has spread to the Middle East, Africa and the Mediterranean. In 1992 RPW was first detected in Egypt [2] then spread through the Northern Mediterranean Basin [3–5], where it attacked the highly sensitive ornamental palm *Phoenix canariensis* [6]. More recently RPW has been detected in Australia, China, Japan and the Caribbean [7–12]. Globally, the pest has a wide geographical distribution in diverse agro-climates and an extensive host range in Oceania, Asia, Africa and Europe [1]. The RPW life cycle, from egg to new-born adult, occurs in the palm tree trunks [13] in which the weevil feeds on tissues and sap. Larvae develop inside palm trees resulting in the destruction of palm tissue leading, eventually, to the death and collapse of the tree. The palm tree trunk tissues consist of more than 80% (wt. %, dry basis) of cellulose, hemicellulose and lignin [14]. For this reason, they represent a non-easily digestive substrate for most eukaryotes.

The role played by mutualistic bacterial consortia supporting their insect host with essential compounds missing from the diet (i.e. amino acids, vitamins and cofactors), or by contributing to the digestion of the ingested material, is well documented [15–20]. In the cases of aphids and cicadellids, these contributions are provided by intracellular bacteria (respectively, *Buchnera aphidicola* and ‘*Candidatus* Sulcia muelleri’ [21–24]), while in termites and other insects, essential compounds are provided by complex microbial communities, in some cases including both intracellular bacteria and gut microbiota, e.g. coackroaches [15,25,26].

Considering the economic and social impact of RPW, the interest in this pest has significantly increased in recent decades. Most studies have focused on the efficacy of different chemical and bio-control strategies [27,28]. Conversely, little attention was paid to the microbial community associated with RPW, although an intracellular primary endosymbiont has been described in weevils and classified as ‘*Candidatus* Nardonella’ [29–32]. Regarding the gut bacterial community associated to RPW, only preliminary studies have been conducted [33–36], in some cases addressed to cultivable bacteria in order to identify potential insect pathogens useful in bio-control strategies [34,37]. Aerobic and facultative anaerobic bacteria (*Bacillus* sp., *Salmonella* sp., *Enterococcus* sp. and *Xanthomonas* sp.) and bacteria able to degrade polysaccharides and sucrose through hydrolase activity (e.g., *Klebsiella pneumoniae* and *Lactococcus lactis*) have been discovered and isolated from the RPW’s gut [33,35]. To date, no studies have investigated the existence of stable core-microbiota that may be useful for the development of efficient bio-control strategies [38,39].

The main aim of the present study is to investigate the influence of the environment (mainly food sources) in shaping the microbial diversity of RPW by *i*) comparing the microbiota of RPW individuals collected on palm tissues *vs* laboratory individuals reared on apple-based substrate; *ii*) comparing the endophytes of palm tissues with the weevil’s microbiota and *iii*) evaluating the metabolic potential of the identified microbial consortia from individuals collected on palm tissues *vs* laboratory ones. Moreover, the bacterial cultivable fraction associated to laboratory-reared individuals was estimated by the use of several isolation media.

## Materials and Methods

### Ethics statement

*Rhynchophorus ferrugineus* and its sister species *R*. *vulneratus*, the most damaging insect pest of palms in the world, are not listed in any national or regional law as protected or endangered species. The collection of specimens in Malaysia was made in the private properties of cooperating landowners. The collection of specimens in Italy was not subjected to any restriction, moreover the specimens sampling occurred in not protected areas and under the surveillance of Servizio Fitosanitario della Regione Sicilia.

### Specimens sampling, dissection and DNA extraction

Male and female RPW adults, and host plant tissues were collected on *Phoenix canariensis* in Catania, Italy (S1 Table). Among the insects, three individuals (one male and two females) were preserved in absolute ethanol, whereas six additional specimens (three males and three females) were transported to laboratory and maintained alive on diets of apple for four weeks with a natural light-dark cycle (14:10), RH = 65% and temperature = 28°C. Three adults of *Rhynchophorus* sp., firstly identified as *R*. *ferrugineus*, were collected using specific traps baited with the aggregation pheromone Rhyfer 220 (Intrachem Bio Italia S.p.A.) in Genting Sempah, Selangor, Malaysia (S1 Table). All the collected specimens were immediately stored in absolute ethanol, furthermore, considering the size of the specimens 1 ml of absolute ethanol was injected with sterile syringes in each specimen. Before dissection, samples were surface sterilized following the protocol reported in Montagna *et al*. [40]. Specimens, after anaesthetisation at -20°C, were dissected aseptically using sterilized scalpels and forceps under a Leica MS5 stereomicroscope. The insect content, including the whole gut, the fat body and reproductive system, was removed and homogenised for the DNA extraction. Palm tissues were dried at room temperature before DNA extraction. DNA was extracted using DNeasy Blood and Tissue Kit (Qiagen), for the animal tissues, and the DNeasy Plant Kit (Qiagen), for the palm tissues, following the manufacturer’s instructions in both cases. The final elution was realised in 300 μl of AE buffer and DNA was quantified by spectrophotometry.

In 2013 Rugman-Jones *et al*. [41] reported the presence of two *Rhynchophorus* species in Malaysia: *R*. *ferrugineus* and *R*. *vulneratus*, which have been in synonymy until the publication of the cited work. Since the identification at species level starting from morphological features is sometimes impossible, we performed molecular identification of the three specimens collected in Malaysia. The following strategy has been adopted: total genomic DNA was extracted from three individuals sampled at Genting Sempah (Malaysia) and used as template to amplify a 5′ upstream region of the cytochrome c oxidase subunit I gene (*coxI*) from the mitochondrial genome. The PCR cycle was performed using the Folmer’s primers LC01490 and HC02198 [42]; complete details of the laboratory procedures are reported in Rector *et al*. [43]. The acquired sequences, deposited at the European Nucleotide Archive with accession numbers LN612634-LN612636, were screened for identification by a blast search over the National Center for Biotechnology Information (NCBI) GenBank nucleotide collection using the Mega BLAST procedure [44] available at its website (http://www.ncbi.nlm.nih.gov/blast). Our query sequences were unequivocally assigned to *R*. *vulneratus* with high sequence identity in all cases (Identity 99–100%, 0 gaps).

### Pyrosequencing and data analysis

Pyrotag assays were carried out using bacterial universal primers (27 F mod 5’—AGR GTT TGA TCM TGG CTC AG—3’; 519 R mod bio 5’—GTN TTA CNG CGG CKG CTG—3’) targeting the variable regions of 16S rRNA V1–V3 and amplifying a fragment of approximately 400 bp. The amplified 16S rRNA regions contain enough nucleotide variability to be useful in identification of bacterial species [45,46]. Primers were modified by the addition of a GS FLX Titanium Key-Primer AGR GTT TGA TCM TGG CTC AG and a multiplex identifier (MID) sequence specific to each sample. The MID sequences (forward) were reported for the respective weevil specimen in S1 Table. PCR reactions and next generation 454 pyrosequencing were performed commercially (MR DNA, Shallowater, TX—U.S.) as described in a previous work [40].

A total of 345973 raw, barcoded amplicons of the V1–V3 region of the 16S rRNA gene, were obtained. The reads were trimmed to remove pyrosequencing adaptors, low quality base calls (<30 Phred score) and size-selected (between 350 and 500 bp) using the QIIME [47] pipeline filtering scripts. The total of 138738 high quality sequence reads that were not flagged as chimeras after screening with *Chimeraslayer* were clustered into operational taxonomic units (OTUs), based on a sequence identity threshold of 97%, using *Uclust* [48]; drawing one sequence for each OTU, as representative, and then aligned to Greengenes (http://greengenes.lbl.gov/) using PyNast [47]. Sequences representative of each OTU were taxonomically classified by BLASTn-based comparisons to the *Greengenes* and *Silva* databases within QIIME. The resulting set of OTUs was used in diversity analyses (see below). The analyses were carried out using the various scripts of the QIIME pipeline.

The 16S rRNA gene sequences obtained by 454 pyrosequencing assays were deposited in European Nucleotide Archive with accession numbers PRJEB6918.

### Diversity and statistical analyses

The diversity indices and the following analysis (exceptions are specified) were estimated using the vegan-package “Community Ecology Package: Ordination, Diversity and Dissimilarities” [49] in the R software package (R Project 3.0.2; http://cran.r-project.org/). The Shannon *H* index [50], Pielou’s evenness [51] and total species richness index Chao 1 [52–54] were estimated. The significance of the differences between the analysed statistics (i.e. the number of identified OTUs within each specimen, the Shannon *H* diversity and Pielou’s evenness indices) were tested with the non parametric Kruskal-Wallis tests [55] after the assessment of the equality of variances adopting Levene’s test [56]. In case of comparison between to two groups the Mann-Whitney test was adopted [57]. These tests were performed using lawstat-package [58] in the R software package.

The β-diversity matrix was computed using the script *beta_diversity*.*py* implemented in QIIME [47] and UniFrac [59], which uses as input the OTU table with the amount of the observed 16S rRNA sequences for each OTU for each weevil and the phylogenetic tree constructed using FastTree [60]. Since the purpose of our ordination analysis was to reveal significant pattern of variation in the microbiota composition between the different specimens, the unweighted unifrac metric was adopted [61]. The obtained matrix was used as input for the Principal Coordinates Analysis (PCoA [62]).

The OTU table, containing the abundance of sequences clustered within each identified OTU for all of the processed insect specimens, was transformed into a presence-absence matrix in order to be processed for further analyses. The similarity between the microbial communities associated with the three groups of specimens (i.e. RPW from wild population, RPW from wild population and reared on apple for four weeks and the sister species *R*. *vulneratus*) was analysed through a hierarchical cluster analysis. This analysis was conducted using the function *hclust* in R stats-package (R Project 3.0.2; http://cran.r-project.org/). The dissimilarity matrix, used as input for the hierarchical cluster analysis, was estimated by *vegdist* using the Bray and Curtis dissimilarity index [63]. In addition, to test the reliance of the obtained results the same analysis was also performed on the dissimilarity matrix obtained adopting Jaccard [64] and Kulczynski indices. In order to test the significant dissimilarity between the microbiotas associated to the groups identified by the clustering analysis (corresponding to RPW_PALM_, RPW_APPLE_ and R_VULN_), the dissimilarity matrices were subjected to a nonparametric one-way analysis of similarity (ANOSIM [65]) as in a previous work [66]. In order to estimate the change in species composition between the bacterial communities harbored by RPW_PALM_ and by RPW_APPLE_, two components of β-diversity (i.e. species turnover and nestedness) were estimated with the R package *betapart* [67] using Simpson’s dissimilarity index as in Montagna *et al*. [40]. To investigate the common OTUs present in the three groups of weevils, an analysis of commonality was performed and visualized through a Venn diagram using the *gplots* package in R.

The impact of ecological traits (i.e., the food source and the temperature at which the samples live) on the bacterial communities associated with insects has been evaluated by correlation with results of the OTU-table Non-Metric Multi-Dimensional Scaling (NMDS [68]). The adopted procedure has been described in a previous study [40]. Concerning the diet, two substrates were considered: the palm tissues and apple; while, regarding the temperature two classes have been adopted based on the monthly average temperature at the time of the sampling: 20°C for specimens collected in late October, 2012 in Catania and, > 25°C for specimens maintained in lab and for specimens collected in Malaysia in January, 2012.

### Predictive functional profiling

To explore the functional profiles of our bacterial community data set, we used PICRUST (Phylogenetic Investigation of Communities by Reconstruction of Unobserved States http://picrust.github.com, 3 July 2013 [69]). For the analysis, OTUs were closed-reference picked against the 18 May 2012 Greengenes database using QIIME v 1.6 according to the online protocol. We predicted the bacterial metagenome for each of our samples. The accuracy of metagenome predictions was measured by the Nearest Sequenced Taxon Index (NSTI), with lower values indicating a closer mean relationship [69]. Our samples had NSTI values of 0.06 ± 0.02. For comparison, Langille *et al*. [69] found that human-associated samples had the lowest (best) NSTI values (0.03 ± 0.2), while communities such as soil had a much higher NSTI value (0.17 ± 0.02). The table with the predicted gene family counts per-samples according to Cluster of Orthologous Groups [70] and identifiers adopted by KEGG Orthology [71] was cleaned removing: *i*) all categories not related to the bacterial physiology/metabolism in a symbiotic perspective; and *ii*) categories with count equal to 0. Statistical analyses (i.e. Levene’s and the non parametric Kruskal-Wallis tests) were performed among the weevil groups in order to account for the differences in the amount of counts.

### Bacterial isolation

Since most of the strategies adopted in biocontrol programs (e.g., the sterile insect technique or the use of bacteria as biocontrol agents) are based on insects’ rearing, isolation trials were carried out in order to investigate the bacterial cultivable fraction associated to RPW_APPLE_, which may be the target of such strategies. Two adults (one male and one female) sampled in Catania, Italy, and reared under lab condition on apple for 30 days (see previous paragraph for details on the rearing condition), were dissected to obtain gut, ovaries, testes, and the female hemolymph. Each organ was smashed in 500 μl 0.9% NaCl and serial dilutions were plated on agarized LB, R2A, TSB and PDB media, supplemented with 100 μg/ml cycloheximide, and incubated in aerobic conditions or in microaerophilic Gaspak at 30°C. After isolate purification, the bacterial collection was de-replicated by the analysis of internal transcribed spacer (ITS)-PCR and one or two representatives of each ITS group were identified by partial sequencing of 16S rRNA gene after DNA extraction and amplification with the primers 27F and 1492R [72]. Partial 16S rRNA gene sequences were deposited in the European Nucleotide Archive under the accession numbers LN623577-LN623640.

## Results

### Bacterial diversity associated with weevil (α-diversity)

A total of 138,738 bacterial 16S rRNA sequences have been obtained from the 12 RPW samples (median = 7599.5). The coverage of microbial α-diversity associated with each specimen was investigated through visual analysis of the rarefaction curves (α-diversity indices and observed species plotted *vs* simulated sequencing effort; S1, S2 Figs.). Using the Shannon index as a metric to measure α-diversity, all samples reached a plateau at value of ~1600 sequences per samples (except for a female specimen reared in lab for which only 1670 high-quality 16S rRNA gene sequences have been obtained), indicating that the microbial α-diversity associated with each specimen was well covered.

Table 1 reports the values of the estimated diversity indices (i.e. Chao-1, Shannon *H* diversity and Pielou’s *J* evenness indices) for the bacterial communities associated with the RPW and *R*. *vulneratus* specimens. The bacterial communities associated with the specimens belonging to the three groups were found to differ significantly in terms of diversity indices (*H*’ χ^2^ = 8.69, *P* = 0.013; *J*’ χ^2^ = 6.85, *P* = 0.032). In particular, the bacterial communities associated with 3 specimens of RPW directly collected from palm are significantly more diverse than that associated with the 6 specimens maintained in laboratory and fed with apple for four weeks (*H*’ U = 18, *P* = 0.024; *J*’ U = 18, *P* = 0.024). Differences were also observed in the total amount of bacterial OTUs associated to the three groups of samples (OTUs χ^2^ = 6.38, *P* = 0.0413). This trend was also observed using the Chao-1 richness estimator (data not shown).

**Table 1:** Diversity indices estimated for the bacterial communities associated with the analyzed *Rhynchophorus* specimens.

Identifier	Group	*N seqs* ^b^	OTUs	H’	J’	Chao-1
I_palm_F1	RPW_PALM_	5420	1060	5.61	0.56	1605
I_palm_F2	RPW_PALM_	5398	1028	5.74	0.57	1652
I_palm_M3	RPW_PALM_	5835	1142	5.69	0.56	1667
	RPW_PALM_ ^a^		1076.7±58.8	5.68±0.07	0.56±0.01	1641.3±32.3
I_apple_F1	RPW_APPLE_	13605	722	4.4	0.46	1082
I_apple_F2	RPW_APPLE_	8635	296	3.03	0.37	485
I_apple_F3	RPW_APPLE_	1670	209	3.98	0.52	345
I_apple_M3	RPW_APPLE_	8292	404	3.62	0.42	606
I_apple_M4	RPW_APPLE_	6907	420	3.67	0.42	617
I_apple_M5	RPW_APPLE_	8764	520	4.25	0.47	707
	RPW_APPLE_ ^a^		428.5±179.4	3.83±0.5	0.44±0.05	640.3±250
MYS_field_F1	R_VULN_	2354	134	2.98	0.42	189
MYS_field_F2	R_VULN_	12106	342	3.54	0.42	473
MYS_field_M3	R_VULN_	59752	633	2.56	0.27	880
	R_VULN_ ^a^		369.7±250.6	3.03±0.49	0.37±0.08	588±347.5

^a^ The mean and standard deviation of the estimated diversity indices are reported for each analyzed group of weevils;

^b^ Number of sequences obtained for each specimens after chimeric and contaminants removal.

Based on the Chao-1 index, approximately 66% of the α-diversity was recovered by our analysis. The microbiotas associated with RPW_PALM_, with *J*’ = 0.56 ± 0.01, resulted more balanced than the bacterial communities associated both with lab-maintained RPWs (*J*’_APPLE_ = 0.44 ± 0.05) and with the sister species *R*. *vulneratus* (*J*’_VULN_ = 0.37 ± 0.08). The communities associated with the latter two groups were dominated by few taxa. In the high-evenness community associated with RPW_PALM_ the dominant OTU accounts for 8.4% ± 2.4 of the insect’s microbiota respect to those explained by the dominant OTUs in RPW_APPLE_ and R_VULN_, in which they made up respectively 15.4% ± 8.1 and 33% ± 12.1 of the total microbial diversity. These results were also visually confirmed by the rank-frequency curves plot (S3 Fig.), in which, for each of the 12 RPW and *R*. *vulneratus* samples, the number of high-quality 16S rRNA sequences clustered into each OTUs is reported. This method allows a visual evaluation of OTU richness and evenness [73,74].

### β-diversity and ecological traits

**Fig 1:**
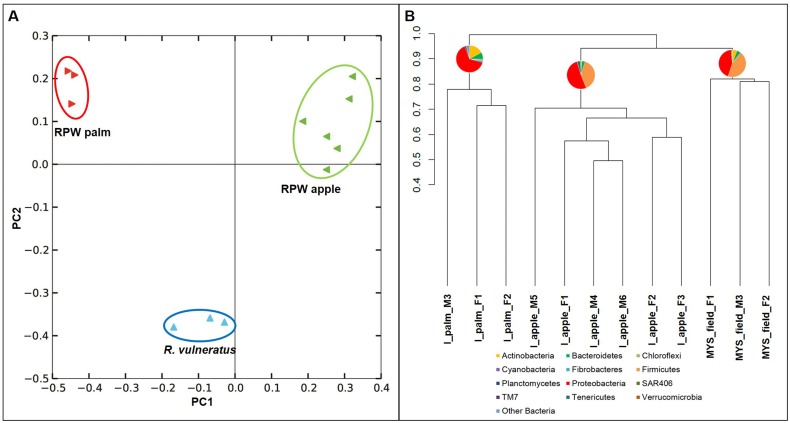
Similarity among the weevil-associated bacterial communities. A: principal-coordinate analysis on the phylogenetic β-diversity matrix obtained starting from the OTU table. The explained variance is as follows: 28.3% 1^st^ component, 14.3% 2^nd^ component. B: hierarchical clustering dendrogram representing the OTU table pairwise dissimilarities between the different analyzed weevils; the pie charts represent the relative abundance of bacterial communities at phylum level.

The β-diversity of bacterial communities associated with the weevil specimens was investigated through a principal coordinates analysis (PCoA) carried on the phylogenetic β-diversity matrix, obtained by UniFrac. The first two components explain a total of 42.6% of the variation (1^st^ component, 28.3%; 2^nd^ component, 14.3%). The analysis revealed an evident clustering of the samples according to each membership group; the first principal component segregates the microbiota of the two groups of *R*. *ferrugineus*, while the second component isolates the specimens of *R*. *vulneratus* (Fig. 1A). The clustering analysis performed on the OTUs’ presence-absence matrix confirms the results obtained by PCoA showing that the pattern of association of the different bacterial community was congruent with the different weevil groups (i.e. the microbiota associated to each specimen clustered together; Fig. 1B). The same results were also obtained by analysing the presence-absence OTUs matrix using Jaccard index (S4 Fig.) and the abundance OTUs matrix adopting the Kulczynski distance (S5 Fig.). These results support the fact that the observed pattern in microbiota composition (i.e. the presence-absence of the different OTUs) is congruent with the grouping factor independently from the bacterial evenness associated with each community. Interestingly, microbiota from specimens of *R*. *ferrugineus* reared in laboratory clustered as the sister group of microbiota from *R*. *vulneratus* specimens.

The estimated β-diversity over the two groups of RPWs, measured as Sørensen’s dissimilarity, resulted in a value of β_SOR_ = 0.982. The two components of the β-diversity, the turnover and the nestedness, resulted in β_SIM_ = 0.973 and in β_NES_ = 0.009, respectively. These values indicate that high OTU turnover and a low nested component have been recovered between the two groups of RPWs, which means that ~97% of the OTUs are different in the two communities. It is interesting to note that after only 30 days of feeding on apple, the bacterial community associated with RPW_APPLE_, derived from RPW_PALM_, dramatically changed compared to the microbiota of the original population, maintaining only a few shared OTUs.

**Fig 2:**
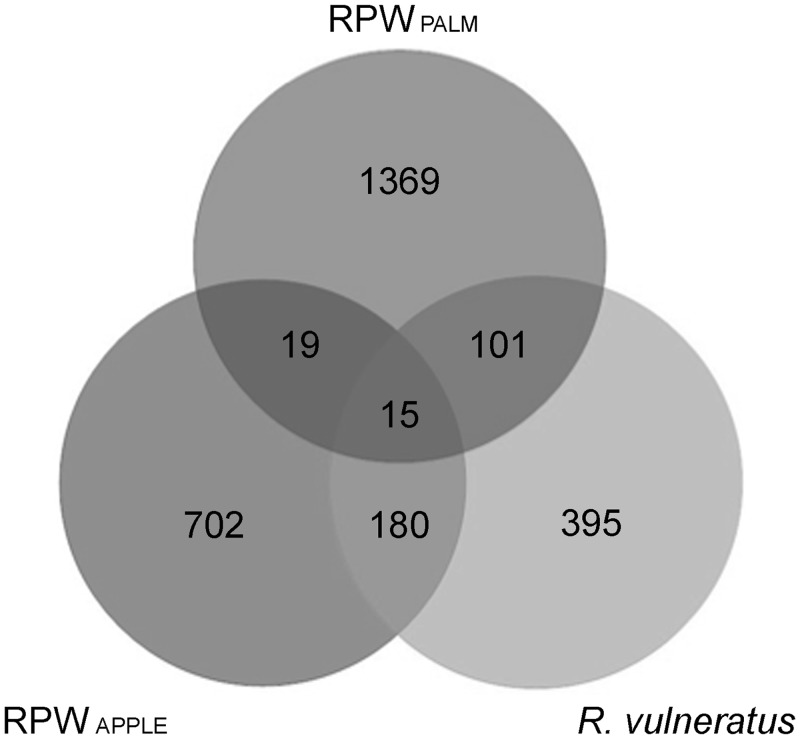
Operational taxonomic units shared by the three groups of weevils. Venn diagram showing the shared bacterial OTUs (at 97% similarity) between all studied weevils groups.

The analyses on the OTUs common to the three groups of weevils showed (Fig. 2) that over a total of 2386 OTUs associated with *R*. *ferrugineus*, 1369 are exclusive to RPW_PALM_ and 702 to RPW_APPLE_. Interestingly only 34 OTUs (19 unique OTUs shared between RPW_PALM_ and RPW_APPLE_ plus 15 unique OTUs common to all) are shared between these two groups of weevils that descend from the same population. Considering all the three groups, only a total of 15 OTUs are shared among them (taxonomic assignment of these OTUs with a comparative analysis with bacterial OTUs isolated from palm tissues are reported below).

**Fig 3:**
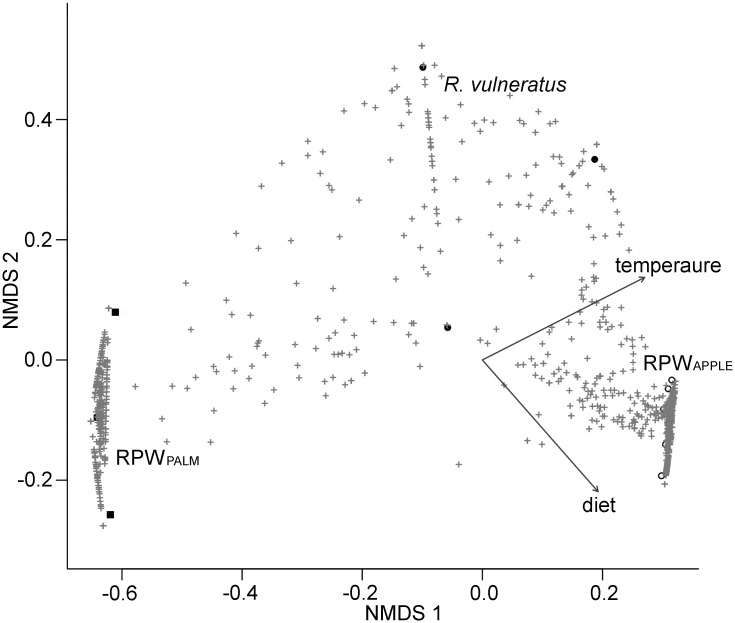
Bacterial communities and ecological factors. Biplot of the first 2 axes for the Non-Metric Multi-Dimensional Scaling representing correlations between the OTUs Chao dissimilarity index and ecological factors (i.e. diet and temperature). The black squares, the black and open circles represent respectively: RPW_PALM_, *R*. *vulneratus* and RPW_APPLE_; while black crosses represent the identified OTUs. The vectors represent the mean direction and strength of correlation of diet and temperature (p-value < 0.05).

Fig. 3 reports the results of the NMDS analysis performed on the bacterial OTU table fitted with both ecological traits: *i*) diet consisting in apple for the lab-reared RPW and palm tissues for field collected RPW and *R*. *vulneratus* and *ii*) the temperatures at which the specimens have been maintained (lab) or are assumed to have developed (wild collected). Both traits significantly explain the dissimilarities among the bacterial communities associated with the three groups of weevils (diet: *R* ^*2*^ = 0.87, *P* = 0.003; temperature: *R* ^*2*^ = 0.94, *P* = 0.006). These results also suggest that temperature may be a confounding factor to explain the impact of different diets on the bacterial diversity and richness associated with the different weevil groups.

### Taxonomic classification of OTUs

**Fig 4:**
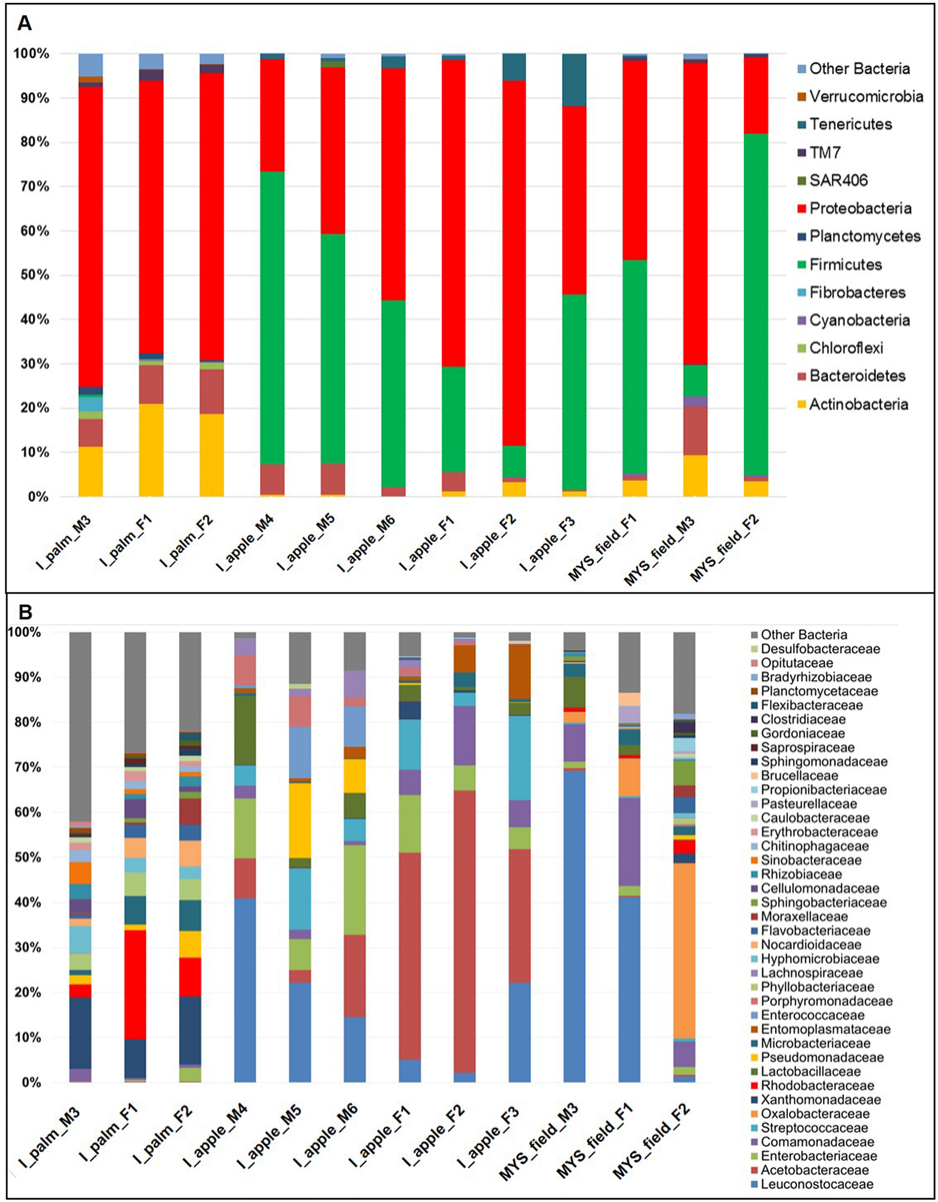
Weevil-associated bacterial diversity. Histogram representing the taxonomic assignment of bacterial 16S rRNA gene sequences associated with the analyzed weevils; A: phylum level, B: family level.

The results of the taxonomic assignment analysis at the phylum and family levels are reported in Fig. 4 (see also S2–S3 Tables). The analysis revealed that the most abundant taxa shared by all the members of the three insect groups belong to Proteobacteria (on average c.a. 64.6% of the sequences in the specimens of RPW_PALM_ group, 51.6% in RPW_LAB_ and 43.3% in R_VULN_; Fig. 4A). The specimens of the groups RPW_APPLE_ and R_VULN_ harbour a second dominant taxon represented by Firmicutes (on average 39.1% and 44.1%), while members of this taxon are scarcely represented in the microbiotas associated to RPW_PALM_ specimens (0.3%). The members of the three groups of insects harbour, with different abundance, also members of Actinobacteria (RPW_PALM_ = 17.1%, RPW_APPLE_ = 1.2% and R_VULN_ = 5.6%) and Bacteroidetes (RPW_PALM_ = 8.2%, RPW_APPLE_ = 3.6% and R_VULN_ = 4.3%). In table 2 are reported the relative abundances of the bacterial genera (with abundance > 1%) associated with the three groups of weevils. Within the *R*. *ferrugineus* feeding on palm the most abundant taxa belong to Xanthomonadaceae (mean 13.2% ± 4.03) and Rhodobacteraceae (mean 11.9% ± 11), to which belong the genera *Luteimonas* (mean 6.6% ± 2) and *Paracoccus* (mean 7% ± 7.8), respectively. In the microbiota associated with RPW_PALM_ specimens, but not with those reared in laboratory, members of the genus *Demequina* (Cellulomonadaceae) and members of Rhodobacteraceae, Phyllobacteraceae and Rhizobiales were recovered. In contrast, the most abundant genus in the bacterial community associated to RPW_APPLE_ and R_VULN_ specimens was *Leuconostoc*, which represents respectively 17.8% (s.d. 14.1) and 37.2% (s.d. 34.1) (Table 2). This genus was not observed in the microbiota of RPW_PALM_ specimens. Other dominant components of the RPW_APPLE_ microbiota are the bacteria of the genera *Acetobacter* (12.9% ± 9.2) and *Lactococcus* (9.3% ± 6.2). Bacteria of the genera *Lactobacillus* and *Entomoplasma* are present in all specimens of *R*. *ferrugineus* reared under lab conditions and in that of the sister species *R*. *vulneratus*, but are not associated with RPW_PALM_. Besides *Leuconostoc*, the dominant bacteria in the *R*. *vulneratus* microbiota belong to the family Comamonadaceae (11.2% ± 7.3) and to the genus *Ralstonia* (Burkholderiaceae). S3–S4 Tables, respectively, report the relative abundances of the bacterial families and of the genera associated with the different weevil samples. In agreement with the results obtained by the biodiversity analysis, the higher number of bacterial families are associated with RPW_PALM_, while samples of *R*. *ferrugineus* reared under lab conditions and those of the sister species *R*. *vulneratus* harbour a lower number of bacterial taxa.

**Table 2:** Genera of bacteria identified in the weevil microbiotas with their relative average abundance expressed as percentage.

	Rhyncophorus ferrugineus			Rhyncophorus ferrugineus						Rhyncophorus vulneratus		
	palm			apple						palm		
	*f* ^a^	*f* ^a^	*m* ^a^	*f* ^a^	*f* ^a^	*f* ^a^	*m* ^a^	*m* ^a^	*m* ^a^	*f* ^a^	*f* ^a^	*m* ^a^
Demequina	2.96	4.13	1.13	-	-	-	-	-	-	-	-	-
Gordonia	0.21	0.55	1.19	-	-	-	-	-	-	-	0.30	0.03
Aeromicrobium	0.74	2.99	1.50	-	-	-	-	-	-	-	-	-
Pimelobacter	0.27	0.94	3.20	-	-	-	-	-	-	-	-	-
Propionibacterium	-	-	-	0.04	0.09	0.24	-	-	-	0.17	2.92	0.03
Dysgonomonas	-	-	-	1.64	0.67	0.06	6.64	6.76	1.92	-	0.50	-
Flavobacterium	-	-	0.09	-	-	-	-	-	-	-	1.55	-
Wautersiella	0.15	1.27	0.96	-	-	-	-	-	-	-	0.17	-
Enterococcus	-	-	0.04	0.24	0.01	-	0.66	11.48	8.97	-	-	-
Lactobacillus	-	-	-	2.60	0.60	1.56	0.99	0.38	0.79	0.04	0.07	0.10
Leuconostoc	-	-	-	5.07	2.17	22.16	40.88	22.15	14.54	41.21	1.37	69.17
Lactococcus	-	-	-	11.05	2.92	18.62	4.49	13.61	4.94	0.25	-	0.35
Clostridium	-	-	-	-	-	-	-	-	-	-	2.30	0.04
Erysipelothrix	-	-	-	1.29	-	-	0.07	0.01	0.41	1.53	0.14	-
Planctomyces	1.01	0.59	0.22	-	-	-	-	-	-	-	0.27	0.04
Asticcacaulis	1.01	0.54	0.33	-	-	-	-	-	-	-	-	-
Devosia	3.68	1.09	1.48	-	-	-	-	-	-	0.17	0.95	0.07
Hyphomicrobium	0.82	1.37	0.69	-	-	-	-	-	-	0.17	-	-
Defluvibacter	0.02	0.06	0.06	-	-	-	-	-	-	-	1.29	0.06
Kaistia	1.11	0.07	0.06	-	-	-	-	-	-	-	-	0.02
Paracoccus	1.03	15.79	4.22	0.04	0.02	-	-	-	-	0.30	1.79	0.25
Acetobacter	0.07	-	-	22.16	23.91	14.79	4.22	1.22	10.95	0.04	-	0.20
Swaminathania	-	-	-	1.07	0.42	0.30	0.19	0.04	0.16	-	-	0.17
Ralstonia	-	-	-	-	-	-	-	-	-	8.37	38.90	2.38
Escherichia	0.03	0.02	0.28	0.06	0.10	-	7.25	-	1.21	0.30	-	-
Gluconacetobacter	-	-	-	0.49	0.16	1.80	0.01	0.41	0.90	-	-	-
Serratia	-	0.02	0.50	1.28	0.19	-	1.19	1.10	2.03	-	1.65	1.39
Trabulsiella	0.02	-	0.31	4.31	0.06	-	0.02	0.52	0.55	1.57	-	0.01
Acinetobacter	0.27	0.46	5.46	0.01	0.01	0.12	-	-	-	0.08	2.07	0.35
Pseudomonas	1.82	1.07	4.41	0.30	-	0.12	-	-	-	0.04	0.26	-
Luteimonas	8.88	4.91	6.00	-	-	-	-	-	-	-	-	-
Stenotrophomonas	-	0.04	0.70	-	0.01	-	-	-	-	0.13	1.64	0.05
Thermomonas	1.68	0.44	1.76	-	-	-	-	-	-	-	-	-

In this table are reported the bacterial genera present with abundance > 1% in at least one specimens. The main differences in the bacterial genera associated with apple and palm are highlighted in bold.

^a^ The gender of the specimens is reported; *f*: female, *m*: male.

Interestingly, no OTUs of the *R*. *ferrugineus* primary endosymbiont “*Candidatus* Nardonella” were recovered in the first analysis of the data using the well-curated RDP database to taxonomically identify OTUs. A more detailed analysis of the OTUs, which were previously identified as unknown Gammaproteobacteria, performed by BLAST against known sequences of “*Ca*. Nardonella” allowed the identification of different OTUs as belonging to this taxon (S5 Table). The identity of these OTUs was later confirmed as the primary symbiont by bidirectional BLAST with values of sequence similarity > 97%. Noteworthy, all the identified OTUs matched with the sequence FJ626262, endosymbiont of *Sphenophorus levis*. The prevalence of “*Ca*. Nardonella” was 100% in all the RPWs and *R*. *vulneratus* specimens. This result confirms the presence of “*Ca*. Nardonella” also in this *Rhynchophorus* species. The fact that the titer of the primary symbiont within the analysed samples was low can be attributed to the fact that the bacteriome and mesenteric caeca, colonized by the primary symbiont [75], represent a small fraction of the total sampled tissues colonisable by bacteria. In addition, the titer of endosymbionts has been demonstrated to vary along the life cycle of their arthropod host (e.g., [76]).

### Bacteria associated with palm tissues

**Fig 5:**
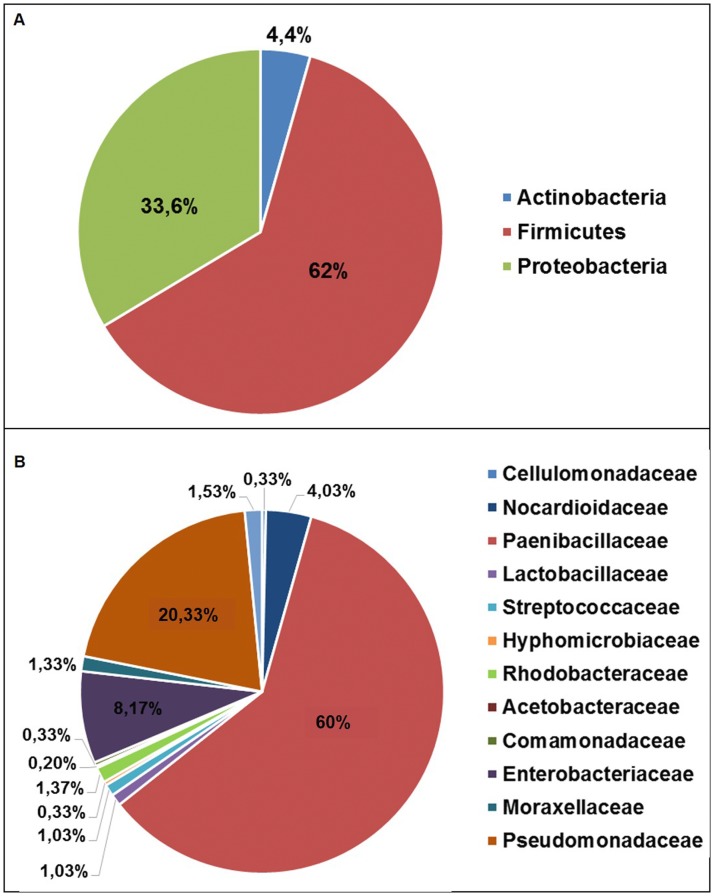
Palm-associated bacterial diversity. Pie charts representing the taxonomic assignment of bacterial 16S rRNA gene sequences associated with the analyzed the palm tissues; A: phylum level, B: family level.

The majority of the 16S rRNA sequences, 60755 out of the obtained 61189 via 454 sequencing from the palm tissues were removed from the analysis, since they matched with the chloroplast. From the three palm samples, a total of 434 16S rRNA sequences were of bacterial origin, and were clustered in 193 ± 78.5 OTUs. The taxonomic composition of the bacterial communities associated with the palm tissues is dominated by members of Firmicutes and Proteobacteria, representing on average the 62% and the 34% of the microbiota (Fig. 5A). The composition of family-level microbiota associated with palm tissues is represented in the pie chart reported in Fig. 5B. The genus *Brevibacillus* (Paenibacillaceae), detected in all the processed palm samples, is the dominant one (average 60 ± 37.7% of the obtained 16S rRNA sequences).

A comparative analysis on the bacteria associated with weevil and palm samples were performed on the genus-level identified OTUs in order to detect any patterns of commonalities. Interestingly, 12 out of the 13 bacterial genera identified in the palm tissues, are recovered, with different degree of prevalence and abundance, also in the weevil microbiota (only *Klebsiella* is exclusive of these tissues). *Brevibacillus*, the dominant bacterium in the palm microbiota, is found to be associated also with two samples of RPW although with a low abundance.

### Metabolic potential

In order to investigate and compare the metagenomic functional potential associated with the different bacterial communities harboured by the three groups of weevils, the 16S rRNA sequences obtained from each specimen were analysed with a dedicated bioinformatics tool (PICRUSt, [69]). In S6 Table are reported the results of the analysis containing the predicted gene family counts per sample for all the categories that have been related to the bacterial physiology/metabolism, in light of a symbiotic relationship, such as the amino acid metabolism, the biosynthesis of secondary metabolites and the metabolism of cofactors and vitamins. The full results of the analysis are reported in S7 Table. Within the cellular processes category, significant differences between all the three insect groups were recovered in bacterial chemotaxis and in bacterial motility proteins (respectively Kruskal-Wallis χ^2^ = 6.54, *df* = 2, *P* = 0.038 and Kruskal-Wallis χ^2^ = 7.27, *df* = 2, *P* = 0.026). In the amino acid metabolism category, significant differences (*P* < 0.05) between the three groups of weevil were reported for Lys, Val, Leu and Ile degradation and for Phe, Trp and β-Ala metabolism. None of the predictions obtained for the category of biosynthesis of other secondary metabolites resulted different based on the Kruskal-Wallis test. Interestingly, other statistically significant differences between-groups were observed in the categories of carbohydrate/energy metabolism (pentose phosphate pathway and nitrogen metabolism) and in the metabolism of cofactor and vitamins (thiamine metabolism). Pairwise comparison carried on the subsets of differentially predicted metabolic pathways, using the Mann-Whitney test, indicated that most of the differences result to be between the *R*. *ferrugineus* that were fed on palms and those fed on apples (p<0.05; S6 Table). From the PICRUSt prediction, these pathways result to be present at a higher percentage in the microbiota associated with RPW_PALM_ than in the one associated with RPW_APPLE_.

### Bacterial isolation from laboratory reared weevils

**Fig 6:**
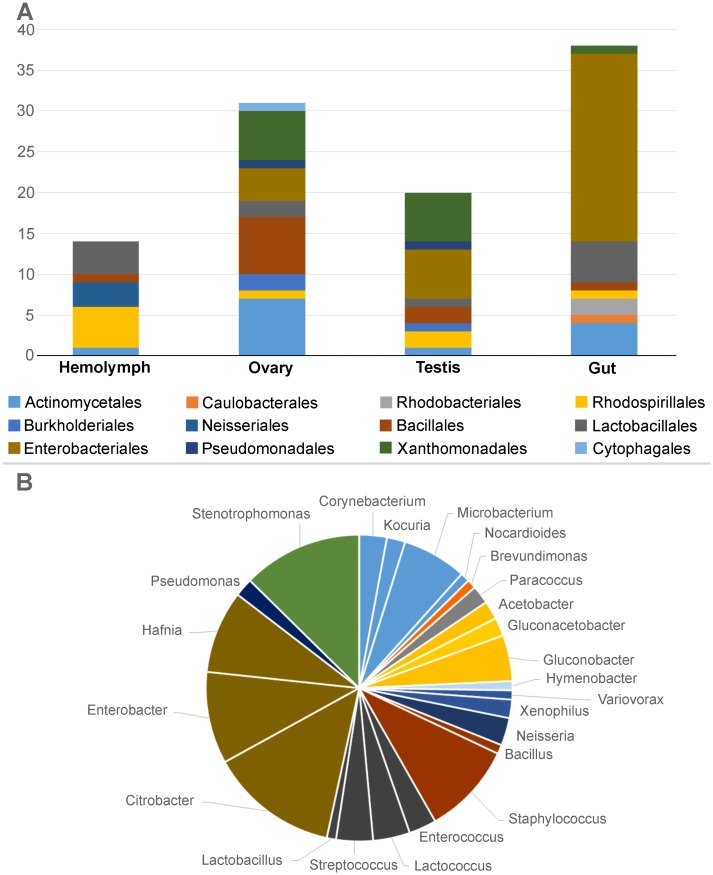
Relative abundance of the strains isolated from the different organs of RPW. A: histogram representing the relative abundance of strains at order level isolated by different organs; B: pie chart representing the cumulative abundance of the different strains at the genus level.

The bacterial cultivable fraction was investigated on RPW_APPLE._. This work allowed the isolation of a total of 103 isolates from different insect organs dissected from laboratory weevils, with the aim to investigate the bacterial diversity associated to the different body districts (S8 Table). Partial 16S rRNA sequencing results showed that the majority of the isolates belonged to Proteobacteria (64%) and Firmicutes (22%, Fig. 6A). Among Proteobacteria, Gamma-subdivision was the most abundant (46% in comparison to Alpha and Beta ones which accounted with 12% and 6%, respectively) with members of Enterobacteriales and Xanthomonadales. Among Firmicutes bacteria belonging to the orders Lactobacillales and Bacillales were isolated. Acetic Acid Bacteria (AAB) accounted for 9% of the total isolates (Fig. 6).

We did not appreciate major differences in the bacterial composition of the different organs and tissues analysed, even though an important fraction of the isolates were obtained from the intestine (no. 38) (Fig. 6B; S8 Table).

The isolated strains belong mainly to the taxa Enterobacteraceae, Lactobacillales, Actinobacterales and Acetobacteraceae. These taxa were also observed in the metabarcoding analysis.

## Discussion

The bacterial communities harboured by the three groups of weevils are dominated by members of Proteobacteria; in addition, RPW_APPLE_ and R_VULN_ specimens harbour a second dominant taxon represented by Firmicutes (see Fig. 4). The unbalanced composition of the bacterial communities associated with these insect groups is reflected also in the Pielou’s evenness index, where RPW_APPLE_ and R_VULN_ show significant lower values that those of RPW_PALM_. In fact, the microbiota associated with RPW_PALM_ is characterized by the absence of dominant bacteria. Most of the RPW_PALM_ exclusive taxa are present with low abundance (<1%); conversely, the microbiota associated with RPW_APPLE_ and R_VULN_ are dominated, with an average abundance of 17.85% and 37.27%, by bacteria of the genus *Leuconostoc*, which is absent in the microbiota of RPW_PALM_. The microbiota of RPW_APPLE_ specimens is characterized also by bacteria of the genera *Acetobacter* (14.47% ±13.6) and *Lactococcus* (9.25% ± 6.2). Interestingly the three groups of weevils shared 15 common OTUs (S9 Table). Among these OTUs, taxa such as *Serratia* and *Ochrobactrum* were observed. Both taxa have been described in association with several arthropod taxa ([40,77,78] *Amblyomma rotundatum*, data not shown). While *Serratia* plays several roles ranging from host protection against parasitoids [79] to the enhancement of host fitness [80], the role of *Ochrobactrum* is yet to be investigated. Noteworthy, three of these shared OTUs belonged to taxa that were present also in palm tree tissues (i.e., one belonging to Comamonadaceae and two to *Stenotrophomonas*).

Culture-dependent methods on organs and tissues of RPW_APPLE_ have allowed obtaining 103 isolates, which were identified by sequencing of partial 16S rRNA gene. Interestingly, a large part of the isolates was constituted by Proteobacteria and Firmicutes, which were present in all the different dissected organs and in the hemolymph. This is in accordance with the data obtained with DNA-based method. In fact, using both cultivation dependent and independent technique, Proteobacteria and Firmicutes members resulted abundant in the laboratory population. Particularly, members of Gammaproteobacteria, with Enterobacteriales and Xanthomonadales representatives, and Firmicutes, with *Lactococcus*, *Streptococcus*, *Enterococcus* and *Lactobacillus*, were retrieved. Bacteria belonging to the genus *Lactococcus* were also previously isolated from wild specimens of RPW [34]. It is interesting to note that members of AAB were isolated from the hemolymph. In insects, these bacteria are generally described as gut associated, but they have been shown to colonize different organs even after administration with food; this indicates that AAB are able to cross the gut barrier and reach other organs, likely through the hemolymph [80–83]. Moreover, in accordance with pyrotag data, laboratory insects harbour also Actinobacteria and Betaproteobacteria, with respectively 13% and 6% of abundance.

The comparative analysis performed on the bacterial consortia associated with the palm tissues with those associated with weevils highlight some patterns of commonalities: 12 bacterial genera out of the 13 identified in palm tissues were also recovered in weevils from the original palm population. Based on this finding, we can hypothesise that these bacteria are ingested by weevils along with palm tissues. Even if we cannot completely exclude that these bacteria are transient components of the weevil microbiota and do not represent stable consortia, the fact that most of them (nine out of 13) are present also in specimens reared on apple for 30 days or in *R*. *vulneratus* suggests that these are stable component of the weevil microbiota. The alternative hypothesis is that palm tissues are contaminated by weevil faeces; the shared insect-palm bacteria would thus represent contaminants from the insects. However, palm tissues examined in this study were healthy and not colonized by the weevil.

Interestingly, even if the microbiota associated with weevils clearly differ among groups in terms of diversity and composition, the predicted metagenome functional potentials related to the bacterial physiology/metabolism in light of a symbiotic relationship were maintained in most aspects. Statistically significant differences among the three groups of weevils were reported for the metabolism of a few amino acids (degradation of Lys, Val, Leu and Ile; metabolism of Phe, Trp and β-Ala), in carbohydrate/energy metabolism (pentose phosphate pathway and nitrogen metabolism) and in thiamine metabolism. These biochemical pathways are linked with the recycling of nitrogen and are thus expected to be highly represented in the microbiota of organisms feeding on nitrogen-poor food sources (e.g., palm tissues, apple). The differences in the carbohydrate/energy metabolism could be explained by the high-sugar content in the food resources dispensed to RPWs in laboratory respect to those feeding on the palm tissues. The apple-based diet provided in the laboratory enriched and selected for *Lactococcus* and *Acetobacter*. These bacteria have been observed in association with several other insects that have a sugar-rich diet [72,82].

The taxonomic composition of the microbiota associated with *R*. *ferrugineus* specimens collected in Catania (Italy) clearly differ from those described in a previous study, where a comparable approach was adopted on *R*. *ferrugineus* collected in Al-Hassa Oasis (Saudi Arabia) [35]. In this study, even if a seasonal variability in the gut microbiota was observed, the dominant bacteria in adult specimens belonged to the genera *Lactococcus* and *Acinetobacter*, whereas *Klebsiella* and *Lactococcus* were detected in larvae. In our study, bacteria of the genus *Lactococcus* were recovered in specimens reared in laboratory on apple, while members of the genus *Klebsiella* were only recovered in the palm tissues. The last findings lead to the hypothesis that the pattern found by Jia and colleagues [35] could result from the effect of the bacteria transmitted to the weevil by the palm tissues.

The bacterial communities associated with the three groups of weevil under study (*R*. *ferrugineus* from wild population, *R*. *ferrugineus* from wild population reared in laboratory for four weeks feeding on apple, and *R*. *vulneratus* from Malaysia) were significantly different. The specimens belonging to *R*. *ferrugineus* of the invasive population collected in Catania harboured the highest number of bacterial OTUs (OTUs = 1077), while a significant decrease in the number of harboured OTUs has been observed in the specimens reared in laboratory (OTUs = 429). This reduction has been observed after a relatively short time of maintenance (30 days) under stable environmental conditions (temperature, humidity, light-dark cycle and food resources) feeding on a sugar-rich resource as the apple. The number of OTUs identified in the sister species *R*. *vulneratus*, collected in the *Rhynchophorus* native area, was lower (OTUs = 370) respect to those of the two groups of *R*. *ferrugineus*. Interestingly, the number of OTUs associated with adult specimens from Saudi Arabia and to larvae reared under hot condition (i.e. 32°C) resulted of ~ 400 [35], a value comparable to those obtained for the RPW_APPLE_ and of *R*. *vulneratus* from Malaysia, but not with the recovered value for RPW_PALM_ and larvae reared at 20°C (1077 and 1049, respectively). We cannot exclude that the differences in OTU number between our specimens and those from Saudi Arabia [35] are due to differences in the used 16S rRNA regions. The number of OTUs detected in the analysed weevils was higher in respect to those observed in other Coleoptera [40,84–86]. Moreover, the composition of the bacterial community associated with the three groups of weevils, which have been analysed through PCoA and ANOSIM, resulted statistically different. These results have also been confirmed by the analysis on the two components of the β-diversity, which was performed on the bacterial community associated with the two groups of RPW. The hierarchical clustering grouped the bacterial community associated with RPW_APPLE_ together with those associated with the sister species *R*. *vulneratus* instead of with those harboured by the co-specific specimens from wild population.

Based on the achieved results on the bacterial OTU diversity, integrated with those obtained by Jia and colleagues [35], we can hypothesize that high temperatures (as those of rearing facilities, of Saudi Arabia and of Malaysia) have caused a decrease in the level of bacterial diversity associated with weevil. Conversely, low temperatures (as those experienced by adults collected in Catania and larvae reared at 20°C) increase the bacterial OTU diversity associated with the insect. Both studies confirmed the high plasticity, in terms of turnover, of the microbiota associated with RPW. Based on our results, environmental abiotic factors, such as the temperature, could play an effect in shaping the diversity of weevil’s microbiota. Similar results have been obtained for another group of phytophagous beetles, in which the altitude is related with the structure of the insect’s microbiota [40]. The interpretation of these findings by biological and evolutionary perspectives can be done in the light of the hologenome hypothesis [87], which argues that the real unit under natural selection is the eukaryotic host together with its associated microorganisms. Harbouring a more diverse and evenly represented microbiota, in addition to the capability to acquire new bacterial taxa from the environment, may confer selective advantages to the host in changing environments (e.g., food resources exploitation, capability to survive in polluted environments).

In conclusion, our study shows that: *i*) the bacterial diversity and evenness decrease in a short time when RPW specimens are reared in laboratory under controlled conditions (temperature, humidity, light-dark cycle and food resources); *ii*) the composition of the bacterial community associated with the three weevil groups clearly differs both within the same population (influenced by the diet) and between the considered species; *iii*) most of the members of the bacterial community associated with palm tissues, from which the specimens of *R*. *ferrugineus* were collected, are also present in the insects microbiota; *iv*) bacterial isolation performed on laboratory reared weevils confirmed pyrotag data; *v*) both the present study and the previous one by Jia *et al*. [35] do not identify a fixed microbiota in RPW, suggesting the importance of the environment in shaping it. The knowledge of the bacterial community associated to this important pest, the metabolic potentials exerted by its bacterial partners, the bacterial dynamics in relation to the environment/diet and their distribution and localization, both in palm and insect organs, together with the possibility to cultivate the bacterial symbionts of this insect, could open new interesting perspectives towards the development of novel strategies for the symbiotic control of weevils.

## Supporting Information

S1 Fig Rarefaction curves for total bacterial communities from the different weevil samples at 3% identity cut-off. Here are reported the curves of the Shannon index.
(DOCX)

S2 Fig Rarefaction curves for total bacterial communities from the different weevil samples at 3% identity cut-off. Here are reported the curves of the cumulative number of observed species.
(DOCX)

S3 Fig Log_10_ transformed relative abundance—rank curves for bacterial OTUs detected in the weevil specimens. (DOCX)

S4 Fig Hierarchical clustering dendrogram representing the OTU table pairwise dissimilarities between the different analyzed weevils. Distance matrix was estimated starting from the presence-absence OTU table adopting the Jaccard index.
(DOCX)

S5 Fig Hierarchical clustering dendrogram representing the OTU table pairwise dissimilarities between the different analyzed weevils. Distance matrix was estimated starting from the OTU table, with abundances, adopting the Kulczynski index.
(DOCX)

S1 Table *Rhynchophorus* specimens analysed in the present study by metabarcoding. (XLSX)

S2 Table Identification at Phylum level of the bacterial communities associated with the 12 weevil samples. (XLSX)

S3 Table Identification at Family level of the bacterial communities associated with the 12 weevil samples. (XLSX)

S4 Table Identification at Genus level of the bacterial communities associated with the 12 weevil samples. (XLSX)

S5 Table Bacterial OTUs identified as “*Candidatus* Nardonella”. (XLSX)

S6 Table Predicted gene families inferred on the base of the bacterial 16S rRNA. (XLSX)

S7 Table Predicted gene families inferred on the base of the bacterial 16S rRNA and related to the bacterial physiology/metabolism, in light of a symbiotic relationship. Kruskal-Wallis, Mann-Whitney tests and p-values are reported.
(XLSX)

S8 Table Bacterial isolates from different insect organs dissected from laboratory weevils (XLSX)

S9 Table Bacterial OTUs shared by the three groups of weevils, the presence of these OTUs in palm tissues is reported. (XLSX)
