# Supplementary material for: Effects of the Diet on the Microbiota of the Red Palm Weevil (Coleoptera: Dryophthoridae)
Source: PLoS One. 2015 Jan 30;10(1):e0117439. doi: 10.1371/journal.pone.0117439 (PMC4311986; doi:10.1371/journal.pone.0117439)
Supplement: S1 Fig — Here are reported the curves of the Shannon index. (DOCX) [file pone.0117439.s001.docx]

**S1 Figure.** Rarefaction curves for total bacterial communities from the different weevil samples at 3% identity cut-off. Here are reported the curves of the Shannon index.

**
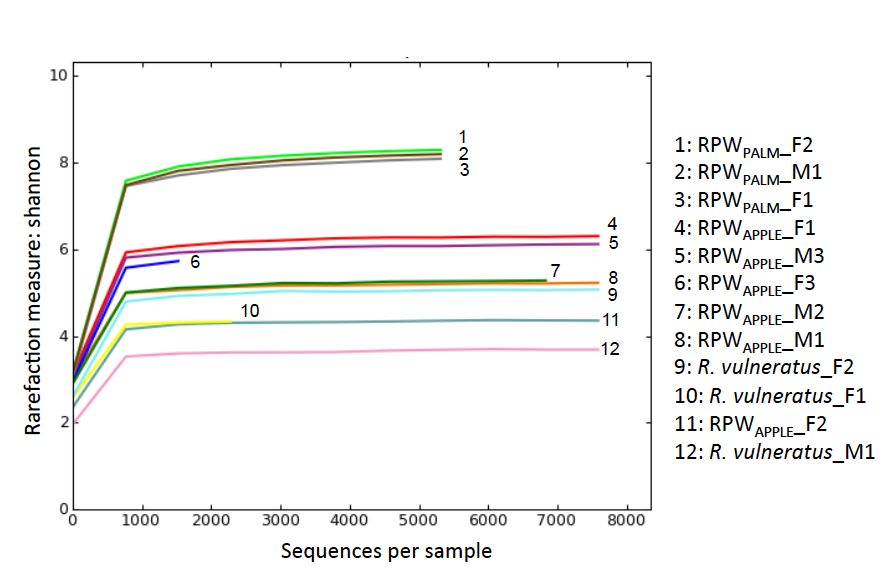
**
