# Supplementary material for: Effects of the Diet on the Microbiota of the Red Palm Weevil (Coleoptera: Dryophthoridae)
Source: PLoS One. 2015 Jan 30;10(1):e0117439. doi: 10.1371/journal.pone.0117439 (PMC4311986; doi:10.1371/journal.pone.0117439)

**S3 Figure.** Log_10_ transformed relative abundance–rank curves for bacterial OTUs detected in the weevil specimens.


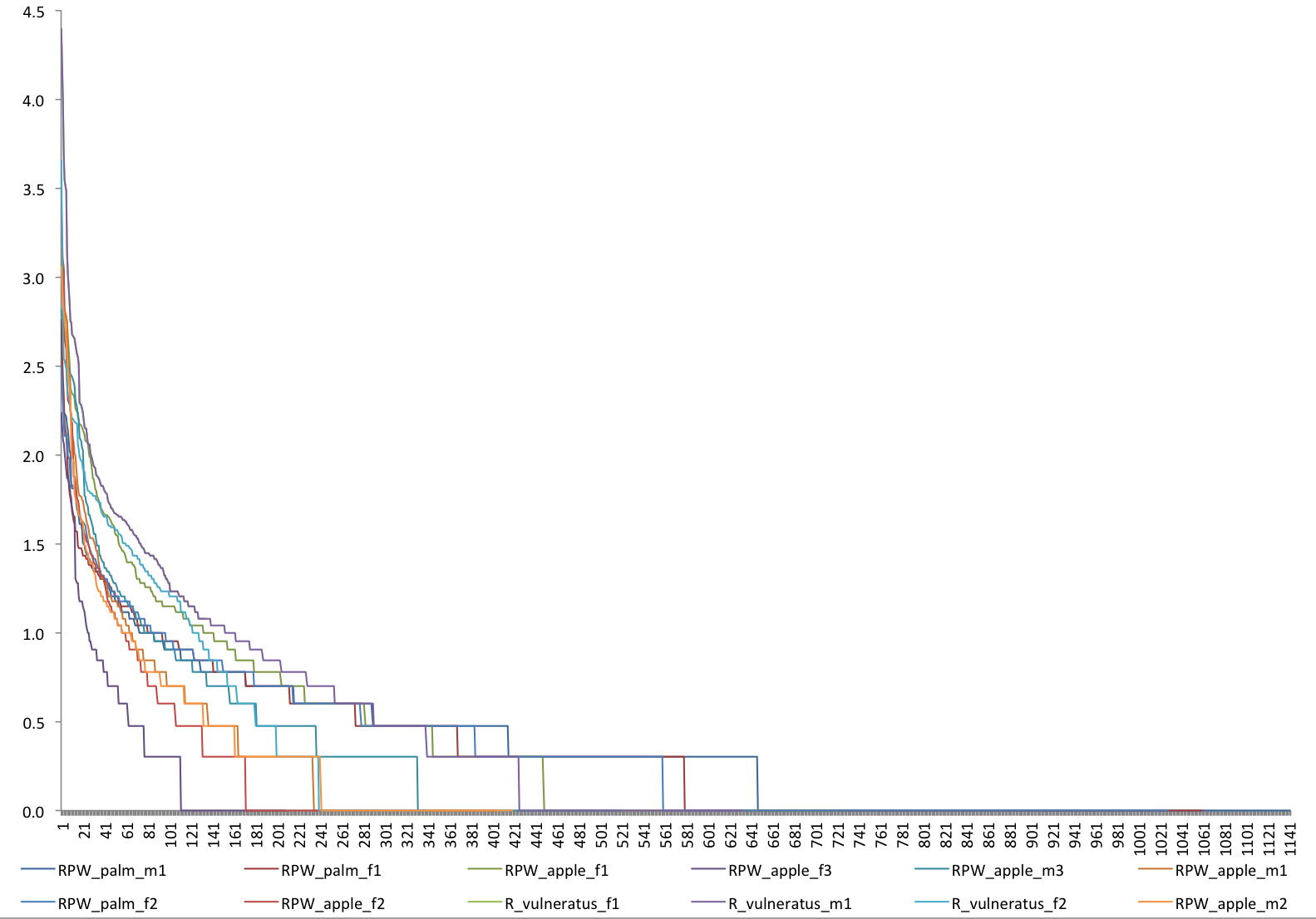

Supplement: S3 Fig — (DOCX) [file pone.0117439.s003.docx]
