# Supplementary material for: Effects of the Diet on the Microbiota of the Red Palm Weevil (Coleoptera: Dryophthoridae)
Source: PLoS One. 2015 Jan 30;10(1):e0117439. doi: 10.1371/journal.pone.0117439 (PMC4311986; doi:10.1371/journal.pone.0117439)

**S5 Figure.** Hierarchical clustering dendrogram representing the OTU table pairwise dissimilarities between the different analyzed weevils. Distance matrix was estimated starting from the OTU table, with abundances, adopting the Kulczynski index.


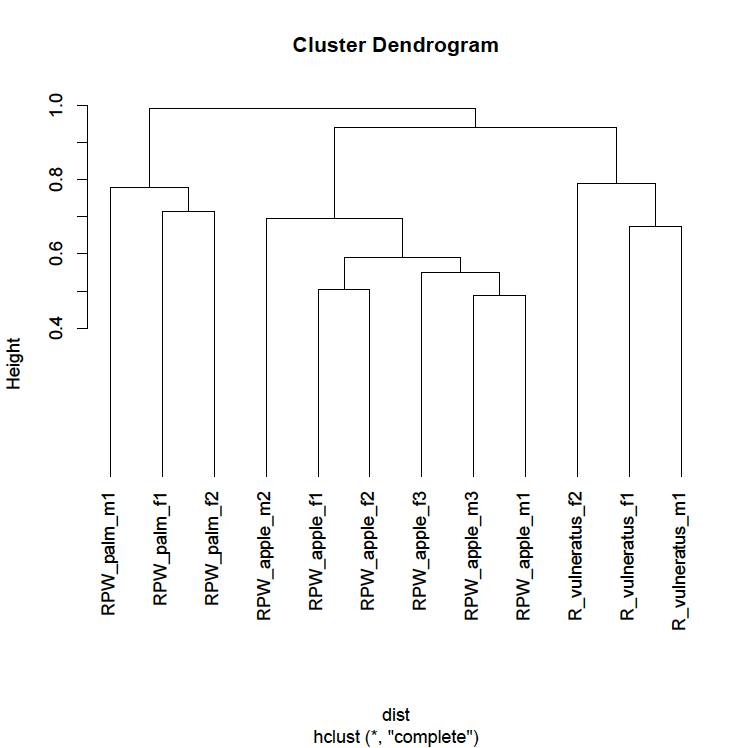

Supplement: S5 Fig — Distance matrix was estimated starting from the OTU table, with abundances, adopting the Kulczynski index. (DOCX) [file pone.0117439.s005.docx]
